# Plasma Metabolic Profiling of Human Thyroid Nodules by Gas Chromatography-Mass Spectrometry (GC-MS)-Based Untargeted Metabolomics

**DOI:** 10.3389/fcell.2020.00385

**Published:** 2020-06-16

**Authors:** Raziyeh Abooshahab, Kourosh Hooshmand, S. Adeleh Razavi, Morteza Gholami, Maryam Sanoie, Mehdi Hedayati

**Affiliations:** ^1^Cellular and Molecular Endocrine Research Center, Research Institute for Endocrine Sciences, Shahid Beheshti University of Medical Sciences, Tehran, Iran; ^2^Department of Agroecology, Aarhus University, Slagelse, Denmark; ^3^Department of Research and Development (R&D), Saeed Pathobiology & Genetics Laboratory, Tehran, Iran; ^4^Department of Chemistry, Faculty of Science, Golestan University, Gorgan, Iran

**Keywords:** thyroid nodules, papillary thyroid cancer, multinodular goiter, metabolomics, GC-MS

## Abstract

One of the challenges in the area of diagnostics of human thyroid cancer is a preoperative diagnosis of thyroid nodules with indeterminate cytology. Herein, we report an untargeted metabolomics analysis to identify circulating thyroid nodule metabolic signatures, to find new novel metabolic biomarkers. Untargeted gas chromatography-quadrupole-mass spectrometry was used to ascertain the specific plasma metabolic changes of thyroid nodule patients, which consisted of papillary thyroid carcinoma (PTC; *n* = 19), and multinodular goiter (MNG; *n* = 16), as compared to healthy subjects (*n* = 20). Diagnostic models were constructed using multivariate analyses such as principal component analysis, orthogonal partial least squares-discriminant analysis, and univariate analysis including One-way ANOVA and volcano plot by MetaboAnalyst and SIMCA software. Because of the multiple-testing issue, false discovery rate *p*-values were also computed for these functions. A total of 60 structurally annotated metabolites were subjected to statistical analysis. A combination of univariate and multivariate statistical analyses revealed a panel of metabolites responsible for the discrimination between thyroid nodules and healthy subjects, with variable importance in the projection (VIP) value greater than 0.8 and *p*-value less than 0.05. Significantly altered metabolites between thyroid nodules versus healthy persons are those associated with amino acids metabolism, the tricarboxylic acid cycle, fatty acids, and purine and pyrimidine metabolism, including cysteine, cystine, glutamic acid, α-ketoglutarate, 3-hydroxybutyric acid, adenosine-5-monophosphate, and uracil, respectively. Further, sucrose metabolism differed profoundly between thyroid nodule patients and healthy subjects. Moreover, according to the receiver operating characteristic (ROC) curve analysis, sucrose could discriminate PTC from MNG (area under ROC curve value = 0.92). This study enhanced our understanding of the distinct metabolic pathways associated with thyroid nodules, which enabled us to distinguish between patients and healthy subjects. In addition, our study showed extensive sucrose metabolism in the plasma of thyroid nodule patients, which provides a new metabolic signature of the thyroid nodule’s tumorigenesis. Accordingly, it suggests that sucrose can be considered as a circulating biomarker for differential diagnosis between malignancy and benignity in indeterminate thyroid nodules.

## Introduction

Papillary thyroid cancer (PTC), which pathologically originates from thyroid follicular epithelial cells, has been globally documented as the most prevalent type of thyroid malignancy, particularly among women (LiVolsi, 2011; Razavi et al., 2019). Ultrasound-guided fine-needle aspiration biopsy (FNAB) is the current preoperative method used for cytological evaluation of PTC and other thyroid nodules (Baloch et al., 2008). Despite its long-standing clinical success, FNAB possesses a major disadvantage: approximately 15–30% of thyroid FNABs cannot cytologically differentiate malignancy from benignity, thus the report would remain as “indeterminate thyroid lesions” (Bongiovanni et al., 2012). On account of this, according to the Bethesda system and a few more reports, the thyroid FNAB must be repeated in these cases to avoid false-positive or false-negative results. However, in some cases and circumstances, the only available option for diagnosis is lobectomy or thyroidectomy (Bongiovanni et al., 2012; Ho et al., 2014). As such, the finding of novel noninvasive diagnostic biomarkers that enable practitioners to distinguish between benign and malignant nodules is a prerequisite to avoid FNAB repetition and unnecessary surgical procedures.

From this point of view, metabolomics-based techniques, in combination with chemometric analysis, are the newest emerging approach, in particular in the cancer areas, which provides an impetus for novel biomarker discovery and to elucidate the molecular mechanism underlying cancer development and progression (Claudino et al., 2012). Experimental studies performed on thyroid cancer reveal the importance of untargeted metabolomics for diagnosis, as well as interpretation of the relationship between clinicopathological features of thyroid cancers with a top-down profile of metabolites in different matrices (Abooshahab et al., 2019). The main focus of previous studies has been on the identification of the metabolites extracted from thyroid cancer tissues or cell lines specimens using nuclear magnetic resonance (NMR) and mass spectrometry (MS), coupled with separation techniques, e.g., gas chromatography (GC) and liquid chromatography (LC) (Yao et al., 2011; Torregrossa et al., 2012; Deja et al., 2013; Wojakowska et al., 2015; Ryoo et al., 2016; Wojtowicz et al., 2017). Some identified metabolites are related to the Warburg effect and glutaminolysis, processes which exclusively occur during tumorigenesis, and which are considered a unique feature of thyroid carcinogenesis (Abooshahab et al., 2019). While previous studies are informative, they are rather limited in introducing the metabolic profile of cancerous cell lines or tissues, thus presentation of a comprehensive metabolic panel of patients with different thyroid lesions remains to be performed.

Obtaining the comprehensive metabolic panel of patients with thyroid nodules could improve the understanding of molecular pathogenesis of thyroid nodules and lead to solving the indeterminate sample problem, through the discovery of new metabolic biomarkers. Blood samples may have more potential in providing a comprehensive picture of metabolic differences between people with malignancy or benignity, compared to tissue samples, as blood is the most frequently used biofluid and contains a high diversity of metabolites with different chemical properties, which provides a snapshot of metabolism in the body. On the other hand, taking blood specimens is a more convenient and less-invasive procedure than biopsy specimens. To date, there have been very few reports with respect to investigating metabolites perturbation in plasma samples of patients with thyroid nodules using the GC-MS technique. Accordingly, this research project aimed to employ GC-MS based untargeted metabolomics in order to characterize the plasma metabolite signatures of patients with PTC, or multinodular goiter (MNG) compared to healthy subjects. As the next step, we explored the probable diagnostic potency of metabolites presented at significantly different levels.

## Materials and Methods

### Study Design and Population

The study was conducted in accordance with the 1964 Declaration of Helsinki and was approved by the Institutional Review Board and Ethics Committee of Research Institute for Endocrine Sciences. Written informed consent was obtained from all participants of this study, including patients and healthy subjects, for the use of their blood samples.

This case-control study was carried out by taking a plasma sample of patients who referred to Shariati Hospital, (PTC and MNG patients) for near-total or total thyroidectomy from November 2015 to August 2016. According to the final surgical pathology of the thyroid reports, only patients with histopathological diagnosis of PTC (but no micro-PTC) and MNG were initially entered into the study. Individuals who had any other types of cancer or metabolic disorders (metabolic syndrome, diabetes, and insulin resistance) were excluded. In total, 19 cases of PTC patients (12 female and 7 male) with a mean age 35.37 ± 10.80 and 16 cases of MNG patients (12 female and 4 male) with a mean age 52.06 ± 10.96 enrolled in this study. The 7th edition of the American Joint Committee on Cancer (AJCC) Tumor-Node-Metastasis (TNM) staging system was performed to determine PTCs staging (Edge and Compton, 2010).

Furthermore, 20 healthy subjects (8 female and 12 male) with a mean age 43.4 ± 14.33 were included in the study as volunteers who referred to the Saeed Pathobiology and Genetics Laboratory for routine checkup tests with the normal range of thyroid-stimulating hormone (TSH) from 0.4 to 4.0 mIU/l. They displayed no thyroid-related disorders, namely hypo/hyperthyroidism, goiter, autoimmune thyroiditis, and thyroid nodules.

About 5 ml of blood was drawn from the pre-operative of each patient (patients were in the primary diagnosis step and received no drug or iodine-therapy before blood collection), as well as healthy subjects in the morning before breakfast, using a plasma-collecting tube containing anticoagulant ethylenediaminetetraacetic acid (EDTA). Subsequently, plasma was separated immediately by centrifugation at 3,000 rpm for 10 min at 4°C. In this way, plasma samples were aliquoted into 1.5 ml Eppendorf microtubes and stored at −80°C until further processing.

### Metabolite Extraction and Derivatization From the Plasma Samples

Plasma metabolites were extracted by adding 1 ml of protein precipitant (methanol/water/isopropanol, 5:2:2, v/v/v) to 50 μl of plasma in 2 ml Eppendorf tubes. The tubes were vortexed, and chilled at −20°C for 20 min, followed by centrifugation at 14,000 rpm for 15 min at 4°C to remove the precipitated protein. Finally, the collected supernatant of each sample was concentrated to dryness using Eppendorf vacuum centrifuge for 3 h at 45°C.

All dried samples were then methoximated and trimethylsilylated with the following protocol. First, 30 μl of a 20mg/ml methoxyamine hydrochloride in pyridine was added to the dried samples, vortexed and placed on a thermo-shaker at 900 rpm for 1 h at 60°C to protect aldehyde and ketone functional groups. This was followed by trimethylsilylation with 60 μl of N-Methyl-N-(trimethylsilyl) trifluoroacetamide (MSTFA) as a silylating agent, vortexed and incubated at 45°C with shaking at 900 rpm for 20 min.

### Untargeted GC-MS Based Metabolomics Analysis

One microliter of aliquot of each derivatized solution was injected at a split ratio of 1:4 into a GC-qMS (Agilent 5975C MSD/Agilent 7890A GC) system equipped with a HP-5ms capillary column (Agilent J&W, 30 m × 0.25 μm × 0.25 mm), at a constant helium flow of 1 ml/min. The inlet, the transfer line, and ion source temperature were set at 280, 150, and 230°C, respectively. All samples were run in a randomized order to avoid systematic bias. The chromatographic method was as follows: 0–1 min at 60°C, 1–22 min ramping to 280°C at 10°C/min rate, 22–32 min at 280°C. Mass spectra were acquired under electron impact (EI) ionization conditions using 70 eV in the mass range of *m*/*z* 50–600. The GC-qMS data were recorded after a solvent delay of 5.4 min.

### Raw GC-MS Data Processing

The acquired data from the GC-MS analysis were pre-processed using Agilent MassHunter qualitative data analysis software for peak picking and mass spectral deconvolution. Subsequently, raw data were exported in CDF format (NetCDF) and then converted to “abf” format to be further processed by MS-Dial software for metabolite annotations (v4.0) (Lai et al., 2018). Automated annotation of metabolites was achieved using MS-Dial’s in-built MS/MS reference libraries. The peak list information of each sample, including average retention time (RT), *m*/*z* values, MS/MS spectra information, and peak intensity (height) were exported by MS-Dial before proceeding to further statistical analysis. Thereafter, the NIST Mass Spectral Search Program (version 2.0) was employed to confirm all metabolite spectra, which was already annotated in MS-Dial against the reference spectrum from the replib, mainlib, and Fiehn libraries with a ≥70% similarity threshold. Lastly, the post-processing was performed on the data, which was previously processed by MS-Dial as follows: all contaminant ions, which were derived from the derivatization reagents, were excluded from the original dataset. In addition, the peak intensity of the duplicate features, which were generated from the same molecule (due to incomplete derivatization), was summed. Subsequently, the metabolite peaks were normalized when performing mTIC method, based on dividing the intensity of each metabolite to the sum of all identified metabolites multiple to the total average mTIC (Fiehn, 2016).

### Statistical Analysis

Significant differences between plasma sample metabolic profiles from PTC, MNG, and healthy subjects were assessed using multivariate and univariate statistical analysis. The variables were normalized by employing cubic root transformation, as well as being scaled by the Pareto scaling method to give equal weight to the variable prior to the data analysis. SIMCA-P 15.0 software (Umetrics, Umeå, Sweden) was employed to construct the multivariate statistic plots such as the principal component analysis (PCA) for data overview and outlier detection, and orthogonal partial least squares-discriminant analysis (OPLS-DA) to determine the metabolic differences between experimental groups. The quality of the models was assessed by the cumulative modeled variation in the *X* and *Y* matrix (*R*^2^ *X* and *R*^2^ *Y*) and the cross-validated predictive ability *Q*^2^ (cum) values. Cross-validated predictive residuals, CV-ANOVA, were used for testing the reliability of the models (Eriksson et al., 2008). Variable importance in the projection (VIP) score values above 0.8 were considered important in this variable for discrimination. A One-way ANOVA and volcano plot were then used to distinguish which metabolites annotated in the GC-qMS dataset were significantly affected by the factor tested in the experiment using MetaboAnalyst, (v4.0).^1^ The *p*-values were adjusted for multiple-testing issues using false discovery rate (FDR) calculations as well. A One-way between subjects ANCOVA was performed to determine the changes in mean intensity of each metabolite in three groups for the effect of age and gender. Heatmap and Box-and-whisker plots were plotted using MetaboAnalyst (v4.0, see footnote 1). Receiver operating characteristic (ROC curve) analysis was applied using IMB SPSS statistics version 26.0 (Chicago, IL, United States) to evaluate area under the curve (AUC) for comparing predictive ability of significant metabolites between tested groups. Graphs for a descriptive analysis were depicted using GraphPad Prism 8.0 (La Jolla, CA, United States) statistical software.

### Metabolic Pathway Analysis

Metabolic pathway analysis was performed using the MetPA tool of MetaboAnalyst (v4.0, see footnote 1), in order to interpret the biological relevance of our findings from both PTC vs healthy groups, and MNG vs healthy groups, which integrates two pathway analyses approaches – enrichment and topology pathway analysis. Likewise, the genes related to the differentially expressed metabolites were identified using the Kyoto Encyclopedia of Genes and Genomes (KEGG) database and Human Metabolome Database (HMDB). This analysis generates a pathway impact score and the associated *p*-value.

## Results

### Clinical Characteristics of the Subjects

The clinical and pathological characteristics of individuals are summarized in Table 1. This analysis included a total of 55 subjects divided into three groups: PTC (*n* = 19), MNG (*n* = 16), and healthy volunteers (*n* = 20). As commonly reported in the literature (Aschebrook-Kilfoy et al., 2013), patients who presented with thyroid nodules were mostly women (PTC and MNG; *n* = 12) than men (PTC; *n* = 7, MNG; *n* = 4). As presented in Table 1 the mean ages of PTC and MNG patients and the standard deviation (SD) were 35.37 ± 10.80 and 52.06 ± 10.96 years, respectively which was significantly different (*p* = 0.000).

**TABLE 1:** Demographic, clinical and pathological characteristic of the study participants.

**Parameter**	**PTC**	**MNG**	**Healthy**
Patient number	19	16	20
**Gender**			
Male	7	4	12
Female	12	12	8
**Age (Mean ± SD; years)**	35.37 ± 10.80	52.06 ± 10.96	43.4 ± 14.33
**Clinical biochemistry tests (Mean ± SD)**	–	–	
TSH (μIU/ml)			1.86 ± 0.97
T4 (nmol/l)			119.038 ± 15.66
T3 (nmol/l)			2 ± 0.33
FBS (mg/dl)			85.47 ± 8.54
**Tumor size (Mean ± SD; cm)**	2.53 ± 1.18	–	–
**Histopathology**			–
Multinodular goiter	19	16	
Classic PTC			
**Extracapsular Invasion**		–	–
Negative	12		
Positive	7		
**Extrathyroidal Extension**		–	–
Negative	19		
Positive	0		
**Invasion^a^**		–	–
Negative	15		
Positive	4		
**Lymph node metastasis^b^**		–	–
Negative	10		
Positive	9		
**TNM stage^c^**		–	–
I	14		
II	2		
III	2		
IVA	1		

*PTC; papillary thyroid carcinoma, MNG; multinodular goiter. ^*a*^Includes blood vascular or lymphovascular or perineurial invasion. ^*b*^Includes N1a and/or N1b. ^*c*^American Joint Committee on Cancer (AJCC) Tumor-Node-Metastasis (TNM) staging system.*

### Untargeted Metabolomics Profiles of Plasma Samples Among Three Groups

**FIGURE 1:**
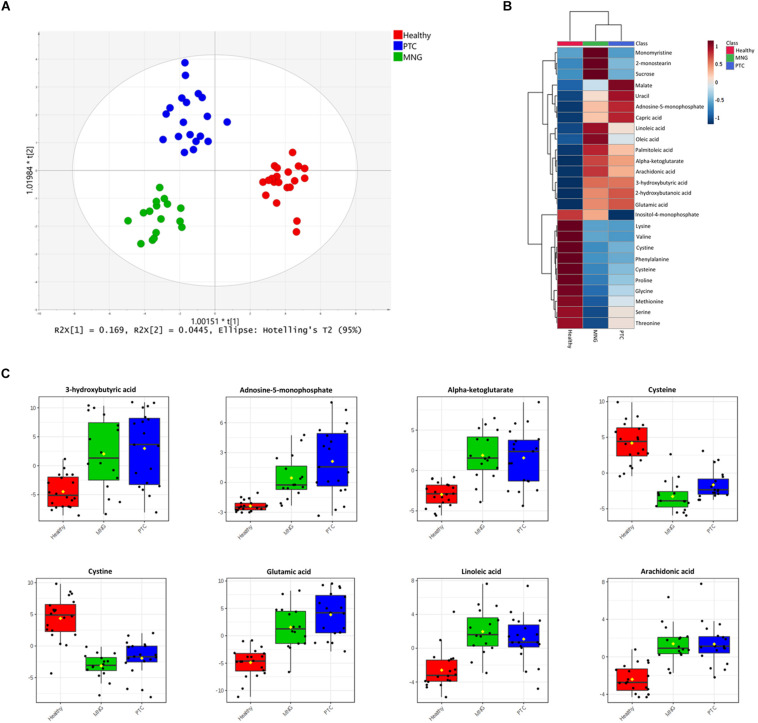
**(A)** OPLS-DA analysis score scatter plots for metabolic profiles of the MNG (green dots), PTC (blue dots), and healthy (red dots) groups showing clear discrimination between the three groups. **(B)** Heatmap visualization of metabolomics data with hierarchical clustering analysis (HCA). Average (mean) intensity was used to indicate the level of difference for single metabolites among three groups. **(C)** Boxplots of the eight most significant metabolites (*P* < 0.05) in the analysis of variance results comparing the three groups (PTC; blue boxes, MNG; green boxes and healthy; red boxes). The *x*-axis shows the specific metabolite and the *y*-axis is the normalized peak intensity. OPLS-DA; orthogonal partial least squares-discriminant analysis, PTC; papillary thyroid carcinoma, MNG; multinodular goiter.

Data processing by MS-Dial resulted in the detection of 776 GC-MS peaks out of which 60 metabolites were structurally annotated and had appearance reliability in all three groups, including the healthy, MNG, and PTC groups. The visualization of the total ion chromatogram (TIC) of the plasma from a healthy human is shown in the Supplementary Figure S1. We initially applied multivariate statistical tools to our data set. First, the PCA showed that the plasma samples from different experimental classes were not grouped separately (Data shown in Supplementary Figure S2). Thus, we performed a supervised OPLS-DA model to determine the metabolites contributing to the discrimination between the PTC, MNG, and healthy groups. The OPLS-DA score plot showed a perfect clustering among the three groups, with acceptable values of predicted variance (*R*^2^*Y* score = 0.87) and predictive ability (*Q*^2^ score = 0.61), indicating that the metabolites were significantly altered in plasma samples of thyroid nodules in comparison with healthy subjects (Figure 1A). The CV-ANOVA test revealed that the model was significant (*p*-value < 0.05) (The models’ parameters are shown in Supplementary Table S1). The goodness of the model was cross-validated by a permutation test (*n* = 100) which showed that the *R*^2^ and *Q*^2^ values of the original model were better than the permutated model’s and indicates of good prediction ability (Supplementary Figure S3).

In addition, One-way ANOVA analysis (*p*-values < 0.05) with Tukey’s HSD test in combination with multivariate analysis, with a VIP score cut off above 0.8 (Data were shown in Supplementary Tables S2, S3), were performed to determine the metabolites which were significantly altered between three groups’ classification (PTC, MNG, and healthy). Our results showed that 28 metabolites out of 60 differed significantly within the compared groups, which persisted for all 28 metabolites (ANCOVA, *p* < 0.05) after adjusting for age and gender (ANCOVA, *p* > 0.05) as a covariate (data not shown). From these data, tricarboxylic acid (TCA) cycle intermediates, amino acids, fatty acids (FAs), and their derivatized, nucleotides etc., were the most significant metabolites distinguished as potential variables which could discriminate healthy subjects from thyroid nodule cases. In plasma samples, 11 amino acids reduced in patients with thyroid nodules compared with those of healthy persons; however, except for the dramatically incremented level of glutamic acid, the reduction pattern of these 11 amino acids was different in PTC than in MNG. Conversely, the metabolites in lipid metabolism, including both long- and medium-chain fatty acids, were elevated in thyroid nodules compared to the healthy subjects. In addition, monomyristine and 2-monosteain (glycerol lipids) and sucrose (carbohydrate) increased significantly in the MNG cases compared to the PTC and healthy subjects. Moreover, the levels of several metabolites including α-ketoglutarate (a keto acid and one of the components of the Krebs cycle), adenosine-5-monophosphate (purine nucleotide), and 3-hydroxybutyric acid (Beta-hydroxy acid) were considerably increased in both the PTC and MNG patients, relative to the control patients. Heatmap representation of the metabolomics dataset with hierarchical clustering analysis (HCA) was performed for a graphical depiction of the metabolites that were altered significantly across the different groups (Figure 1B). In general, these data indicate specific patterns of differences in the metabolites between thyroid nodules and healthy subjects. Box plots comparing the mean intensities for the most significant altered metabolites among three groups is illustrated in Figure 1C.

### Identification of Significantly Altered Metabolites of Plasma Samples Between Two Groups, Separately

**FIGURE 2:**
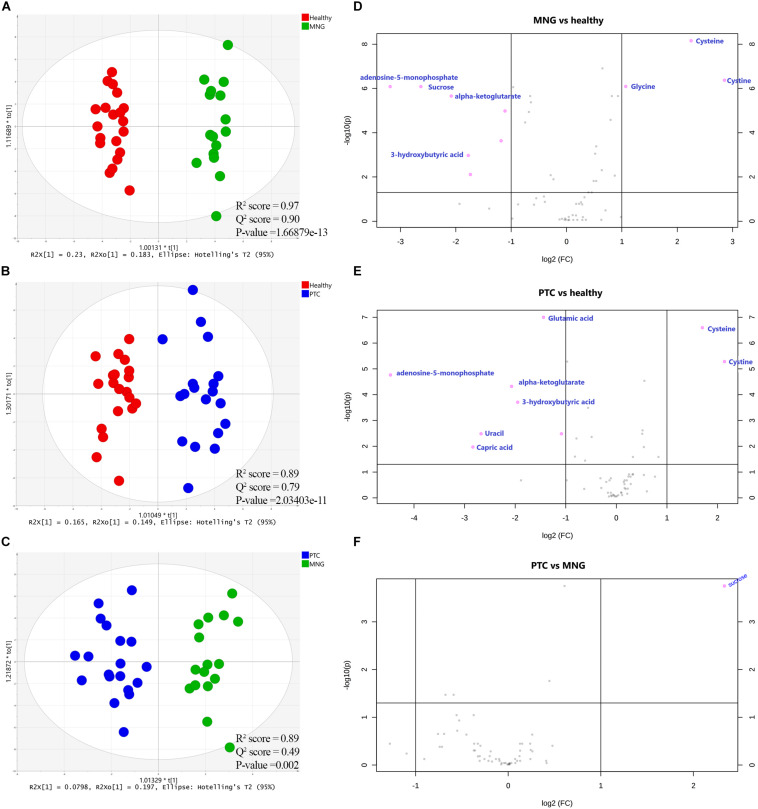
**(A–C)** Supervised (OPLS-DA) analysis score scatter plots illustrating that the metabolic profiles of PTC is distinct from healthy, MNG is distinct from healthy and MNG is distinct from PTC. **(D–F)** Volcano plots of the most significant metabolite changes comparing healthy vs. MNG **(D)**, healthy vs. PTC **(E)**, and PTC vs. MNG **(F).** The most significant differences in metabolites presence; pink dots and the gray dots represent metabolites with no significant differences. The pink dots on the left represent metabolites above the thresholds and their intensities were increased while the pink dots on the right were the opposite. *x*-axis corresponds to log2 (Fold Change) and *y*-axis to −log10 (*p*-value). OPLS-DA; orthogonal partial least squares-discriminant analysis, PTC; papillary thyroid carcinoma, MNG; multinodular goiter.

The first set of analyses constructed the OPLS-DA models in order to achieve maximum separation between two groups individually, including MNG against healthy groups, PTC against healthy groups, and the PTC against the MNG group. As shown in Figures 2A–C, a clear separation was achieved in the score plots for all three models, with acceptable values of *R*^2^*Y* and *Q*^2^. The CV-ANOVA test revealed that the models were significant (*p*-value < 0.05) (parameters of all the models are shown in Supplementary Table S4). The goodness of these models was cross-validated by permutation tests (*n* = 100) which showed that the *R*^2^ and *Q*^2^ values of the original models were better than the permutated models, and indicated good prediction ability (Supplementary Figure S4). The pair-wise comparisons (MNG vs Healthy, PTC vs Healthy and PTC vs MNG) indicated PTC or MNG-specific and common metabolite signatures (Data are shown in Supplementary Figure S5).

**FIGURE 3:**
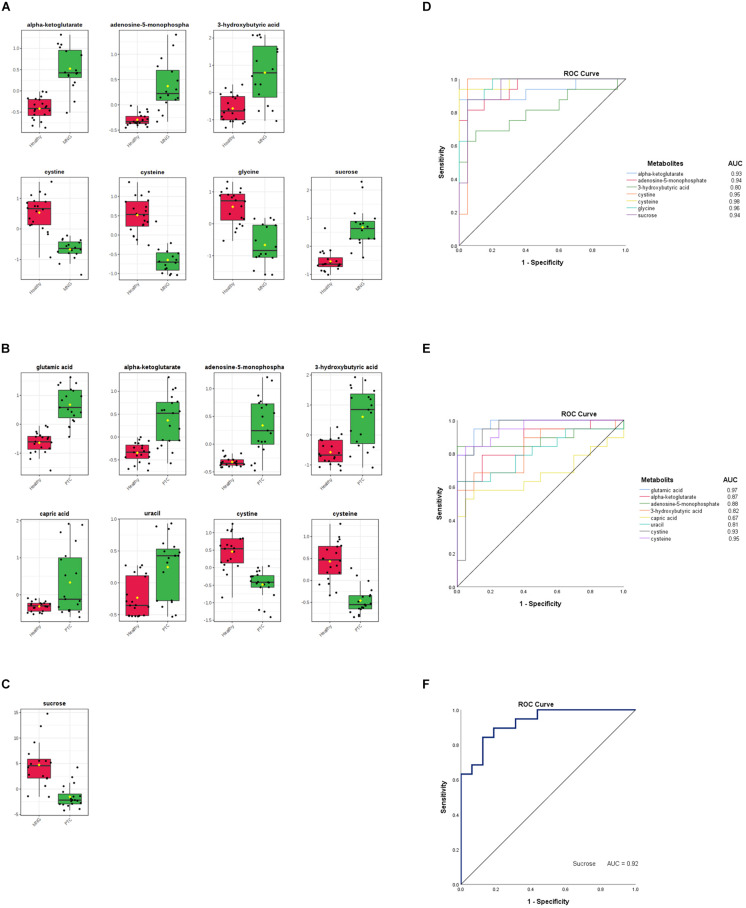
**(A–C)** Box plots visualize each significantly altered metabolite between two groups separately, which were all predicted with the volcano plots. The *x*-axis shows the specific metabolite and the *y*-axis is the normalized peak intensity. ROC curve analyses of the ability of seven metabolites to predict MNG vs healthy **(D)**, eight metabolites to predict PTC vs healthy **(E)**, and one metabolite included sucrose to predict MNG vs PTC **(F)** which were all identified with the volcano plots. ROC; Receiver operating characteristic, PTC; papillary thyroid carcinoma, MNG; multinodular goiter.

In addition, to distinguish the most significant metabolites between two groups separately (PTC vs. healthy; MNG vs. healthy and PTC vs. MNG) univariate volcano plots were performed with fold change (FC) threshold (x) 2 and *p*-value of <0.05 (FDR adjusted *p*-values) which were both log-transformed. The volcano plot from the MNG vs. healthy group (Figure 2D) showed that there were 7 metabolites out of 60 with the most significant changes in cysteine, cystine, glycine, α-ketoglutarate, adenosine-5-monophosphate, 3-hydroxybutyric acid, and sucrose. In the case of the PTC vs. healthy group (Figure 2E), the volcano plot revealed that 8 metabolites out of 60 were significantly changed between two groups, including cysteine, cystine, α-ketoglutarate, adenosine-5-monophosphate, 3-hydroxybutyric acid, glutamic acid, capric acid, and uracil. Furthermore, the volcano plot result comparing the PTC vs. MNG group demonstrated that (Figure 2F), sucrose was remarkably altered in the PTC plasma compared to the MNG. We can see the alterations in the peak intensities of each metabolite between two groups separately using box plots (Figures 3A–C).

Finally, the ROC curve analysis was used to evaluate the diagnostic ability of discriminated metabolites of each volcano plot, as screening biomarkers of thyroid nodules. 1-specificity and sensitivity are located at the *x*-axis and *y*-axis, respectively. The results showed that the AUC of seven metabolites in the PTC vs healthy group (Figure 3D), was larger than 0.81, except for capric acid (AUC = 0.67), AUC of seven metabolites in the MNG vs healthy group was larger than 0.80 (Figure 3E), and AUC for sucrose, a significant metabolite between the PTC vs MNG group, was 0.92 (Figure 3F).

### Differences in Metabolites According to the Clinicopathological Characteristic of PTC Patients

**FIGURE 4:**
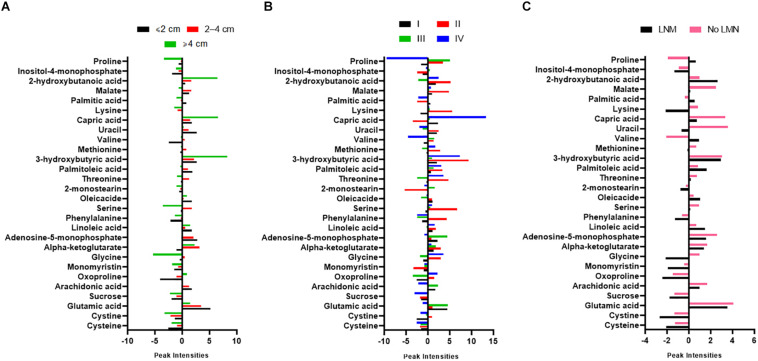
Graphs showing normalized intensity of the 28 significant metabolites according to **(A)** tumor size, **(B)** stages, and **(C)** lymph node metastasis (LNM) of PTC patients. Tumor staging was according to the American Joint Committee on Cancer (AJCC) Tumor-Node-Metastasis (TNM) staging system. PTC; papillary thyroid carcinoma.

A simple descriptive statistical analysis was used to provide the metabolite intensity differences according to the tumor size, stage, and lymph node metastasis (LNM) of PTC patients. As can be seen from Figure 4A, 2-hydroxybutanoic acid, capric acid, and 3-hydroxybutyric acid were increased in patients with a tumor size greater than 4 cm, while serine, glycine, cystine, and sucrose were more decreased in those patients. The capric acid level was more increased in stage IV of PTC patients. Further, glutamic acid and adenosine-5-monophosphate levels were higher in stage III (Figure 4B). In Figure 4C there is a clear trend of decreasing cysteine and cystine, and increasing 2-hydroxybutanoic acid which shows dysregulation of GSH synthesis in PTC patients who have LNM.

### Pathway Analysis

**FIGURE 5:**
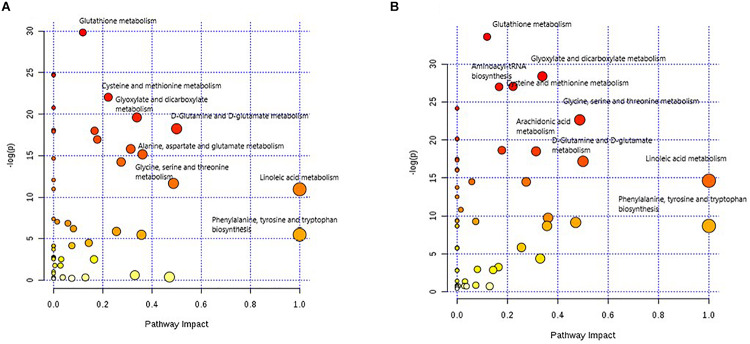
Metabolome view of pathway impact analysis obtained from differential metabolites in PTC **(A)** and differential metabolites in MNG **(B)**. The color and size of each circle is based on *p*-values (yellow: higher *p*-values and red: lower *p*-values) and pathway impact values (the larger the circle the higher the impact score) calculated from the topological analysis, respectively. Pathways were considered significantly enriched if *p* < 0.05, impact 0.1 and number of metabolite hits in the pathway >1. PTC; papillary thyroid carcinoma, MNG; multinodular goiter.

The overview of the pathway impact of attributed metabolites (PTC against healthy and MNG against healthy) was obtained utilizing MetaboAnalyst (v4.0, see footnote 1) (Figure 5). Pathway analysis of altered metabolites shows perturbations in linoleic acid, phenylalanine, arachidonic acid, glycine, D-Glutamine and D-glutamate, and GSH with common influences in both PTC and MNG tumorigenesis (Figures 5A,B). The most influenced metabolic pathway was set as a pathway influence cut off value >0.1 to filter for less important pathways. A detailed pathway analysis table, including all the identified pathways, is presented in Supplementary Tables S5, S6.

## Discussion

For preoperative discriminating of malignancy from benignity in thyroid nodules, FNAB and cytologic evaluation offer a gold standard as a diagnostic tool (Baloch et al., 2008). Unfortunately, up to 30% of evaluated FNABs have been classified as cytologically indeterminate, thus the only way to make a definitive tumor diagnosis is surgery (Bongiovanni et al., 2012). During the past two decades, researchers have been seeking to find a global biomarker capable of improving FNAB diagnostic information using different biological methods such as immunohistochemistry and genetic tests. Several remarkable biomarkers have been reported, including miR- 151-5p, miR-222, galectin-3, thyroid transcription factor 1 (TTF-1), thyroglobulin, calcitonin, carcinoembryonic antigen (CEA), p27, thyroid peroxidase, and BRAF and RAS mutations (Fischer and Asa, 2008; Yu et al., 2012; Ward and Kloos, 2013; Eszlinger et al., 2014; Su et al., 2016; Razavi et al., 2017). However, due to the low sensitivity or specificity rate and the poor positive predictive values of the aforementioned biomarkers, the requirement for employing a non-invasive diagnostic method, as well as finding sensitive and specific biomarkers for early definitive diagnosis of thyroid malignancy is still urgently felt.

Seeking to find diagnostic biomarkers in the -omics era is a promising strategy for solving the indeterminate samples problem. Metabolomics may provide a holistic approach in identifying pivotal metabolites with authentic diagnostic significance (Claudino et al., 2012), however, so far, there have only been five reports of blood metabolomics changes associated with thyroid nodules (Russell et al., 1994; Yao et al., 2011; Shen et al., 2017; Wojtowicz et al., 2017; Huang et al., 2019). Among these previous five studies, only Shen et al. used GC-TOF-MS to identify the serum metabolic signature of distant metastatic PTC (Shen et al., 2017). Therefore, to our knowledge, this is the first report of plasma metabolic profiles of patients with thyroid nodules compared with healthy subjects to identify metabolomics features of thyroid nodules using the GC-MS method. Our data revealed significant alterations in metabolites levels which were mainly associated with sucrose and amino acid metabolism, TCA cycle, FAs metabolism, and purine and pyrimidine metabolism.

The present study showed that the metabolism of about 11 amino acids, including (1) metabolites related to GSH biosynthesis, (2) methionine, (3) glycine, serine, and threonine, and (4) phenylalanine, had been changed in plasma of patients with thyroid nodules compared to healthy subjects. The findings with depleted amino acids in the plasma of patients with thyroid nodules may exhibit excessive consumption of the amino acids by the tumor cells to sustain cell proliferation. The present findings seem to be consistent with other research that found the up-regulation of most amino acids in thyroid carcinoma tissue compared to normal tissue (Wojakowska et al., 2015; Xu et al., 2015). However, the most significant alterations in amino acids, which could discriminate between healthy subjects and thyroid nodule patients, were related to cysteine, cystine, and glycine. Nonetheless, glycine was more decreased in MNG patients. Cysteine and cystine were reduced more significantly in patients with thyroid nodules. The semi-essential amino acid cysteine is a sulfur-containing amino acid which, along with the disulfide bond of cystine, could adopt a variety of oxidation states due to its thiol group (Townsend et al., 2004). Cysteine is a precursor for GSH biosynthesis, which plays a crucial role in sustaining intracellular redox homeostasis by quenching reactive oxygen species (ROS) from mitochondrial respiration. Cancer cells require exogenous cysteine for GSH synthesis to protect themselves from ROS, which is essential for the maintenance of cell proliferation and resistance to cell death (Combs and DeNicola, 2019). A comparison of tissue metabolome profiles between PTC and benign thyroid adenoma (BTA) was performed by Xu et al. (2015) applying GCTOF-MS and LC-Q-TOF methods which showed increased levels of cysteine in both types of tumor tissues. Therefore, decreased plasma levels of cysteine and cystine in patients with thyroid nodules may be explained by the higher consumption of cysteine in the cancer cells. Moreover, we observed elevation in the levels of 2-Hydroxybutanoic acid (2-HBA) which is a metabolite involved in GSH biosynthesis. 2-HBA is a byproduct of the cystathionine cleavage to cysteine in GSH anabolism. The increased levels of 2-HBA along with the decreased levels of cysteine further corroborated the idea of more consumption of cysteine for GSH biosynthesis in patients with thyroid nodules compared to healthy subjects. Since PTC patients with a tumor size greater than 4 cm showed abundant alterations of 2-HBA, it can be assumed that as the tumor size is gradually increased, more glutathione is needed. In addition, PTC patients who had LNM showed the decreasing and increasing pattern of cysteine, cystine, and 2-HBA, respectively. Therefore, it could conceivably be hypothesized that a strong link exists between the perturbation in GSH metabolism and PTC progression.

Another important finding was that there were significant changes in metabolites related to the TCA cycle, particularly glutamic acid and α-ketoglutarate, in patients with thyroid nodules compared to healthy subjects. α-ketoglutarate is one of the important intermediates in the TCA cycle interconvertible with glutamic acid by transamination reaction; therefore, glutamic acid can enter into mitochondria and directly impact energy metabolism *via* the TCA cycle (Mullen et al., 2012). Cancer metabolism documentation noted that glutaminolysis is one of the metabolic properties of cancer cells that promote the conversion of glutamine to glutamic acid in order to maintain the TCA cycle and anabolic process (Mullen et al., 2012; Abooshahab et al., 2019). It can, therefore, be assumed that the increased plasma levels of glutamic acid and α-ketoglutarate observed in patients with thyroid nodules are most presumably due to the glutaminolysis process and the increase in Krebs cycle activity.

In the current results, we noticed major alternations of long- and medium-chain fatty acid metabolism in patients with thyroid nodules. The altered median-chain fatty acid (MCFA) is capric acid (10:0) and the altered long-chain fatty acids (LCFAs) are as follows: palmitic acid (16:0), palmitoleic acid (16:1), oleic acid (18:1), linoleic acid (18:2), and arachidonic acid (20:4). Increased FA β-oxidation is thought to have occurred in patients with thyroid nodules because 3-hydroxybutyric acid, which is an intermediate product of FA β-oxidation, was significantly elevated. All of these findings suggest that increased lipogenesis could be a disrupted metabolic pathway in the molecular pathogenesis of thyroid nodules. One unanticipated finding among the annotated FAs was capric acid (decanoic acid) which was more elevated in patients with thyroid nodules. The PTC patients who were at stage IV or patients with a tumor size greater than 4 cm also showed abundant alterations of this MCFA. It is therefore likely that there is an association between capric acid and thyroid tumor progression. To the best of our knowledge, the capric acid plasma changes in PTC thyroid nodules has not yet been reported in the literature but it is in agreement with Crotti et al.’s (2016) findings which showed an increase in the plasma level of capric acid in patients with colorectal cancer (CRC; Crotti et al., 2016).

The higher plasma levels of metabolites involved in the purine and pyrimidine metabolic pathways such as adenosine-5monophpsphate and uracil were observed in both the PTC and MNG groups. Since purine and pyrimidine nucleotides play an essential role in a large number of cellular processes involving DNA and RNA synthesis, nucleotide cofactors biosynthesis, energy supply, and regulatory mechanisms (Lane and Fan, 2015), it is suggested that the bioenergetics status may be increased in thyroid nodules.

A further novel and interesting finding was that the most changes in the level of sucrose, which is a disaccharide composed of the monosaccharide’s glucose and fructose, were observed in patients with thyroid nodules compared with healthy subjects. It is worth noting that it was the most significantly altered metabolites between PTC and MNG. The previous results, with respect to the various *in vivo* and *in vitro* cancer studies, revealed that the high risk of tumorigenesis was based on high consumption of sugar sweeteners (Wang et al., 2014; Jiang et al., 2016). Since sucrose contains glucose and fructose, a high sucrose sugar diet could promote tumorigenesis *via* some metabolic pathways (Das, 2015). Indeed, in healthy cells, sucrose is broken down into glucose and fructose through a process called hydrolysis. Glucose enters into the aerobic glycolysis pathway which is converted into two molecules of pyruvate. In cancer cells, according to the “Warburg effect,” glucose uptake and aerobic glycolysis metabolism are increased due to the unbridled cell proliferation (Ngo et al., 2015). Consequently, sucrose could amplify the mentioned process by providing one of the most important fuel sources of cancer cells. To date, no one has addressed the impact of sugar-rich diets on thyroid tumorigenesis. As the diet was not controlled in our study, the more we investigate the more we know how thyroid nodules tumorigenesis could be related to sucrose-rich diet intakes.

**FIGURE 6:**
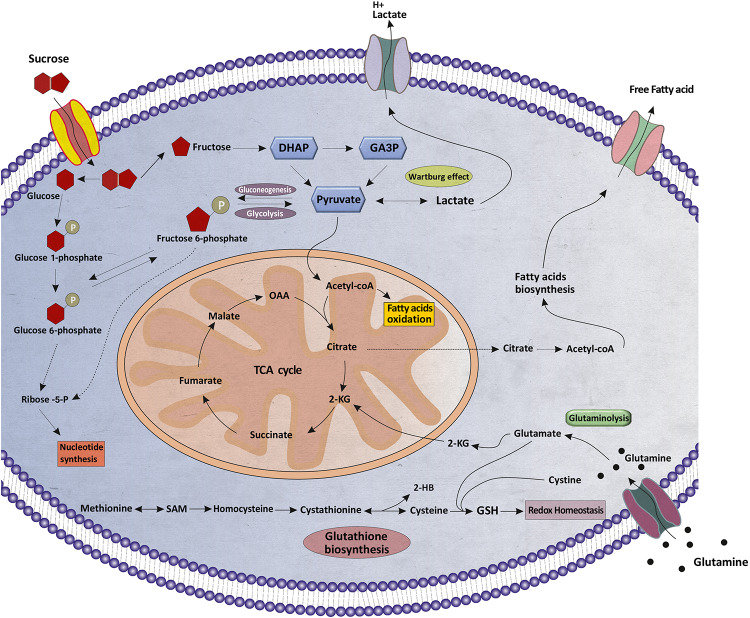
Schematic illustration of the metabolic reprogramming in thyroid nodules. Various aspects of metabolic reprogramming during thyroid tumorigenesis are shown, including sucrose metabolism, fatty acid oxidation, fatty acids biosynthesis, glutaminolysis, glutathione biosynthesis, the TCA cycle, and nucleotide synthesis. α-KG, α-ketoglutarate; TCA, tricarboxylic acid; OAA, oxaloacetate; DHAP, Dihydroxyacetone phosphate; GA3P, Glyceraldehyde 3-phosphate, SAM, S-Adenosyl methionine; 2-HB, 2-Hydroxybutanoic acid; GSH, Glutathione.

An overview of altered metabolites and linked pathways in thyroid nodules obtained from the above mentioned analyses are illustrated in Figure 6. From the results, it is clear that metabolic reprogramming of thyroid nodules, mostly characterized by metabolites, are related to sucrose and glutathione (GSH) metabolism, TCA cycle, FA synthesis, FA beta-oxidation, and nucleotide synthesis.

Although the etiology of the metabolites alterations in thyroid nodularity is quite challenging, there are no sufficient and comprehensive metabolomics studies with respect to the fluctuation of metabolites into the blood stream. Accordingly, it could constitute the subject of future studies that fruitfully explore this issue. Considering that, the decreased or increased plasma level of metabolites could be due to the higher consumption or up/down regulation of them. The comprehensive comparison between the metabolomics profile in the bloodstream and nodular thyroid tissue can provide more detailed insight into the association between the systemic metabolic abnormalities and thyroid tumorigenesis.

## Conclusion

In summary, we performed an untargeted metabolomics investigation to improve our understanding of thyroid nodule metabolism, which could lead to finding novel early diagnostic biomarkers. Metabolism of patients with thyroid nodules, as expected, differs substantially compared to healthy subjects. Metabolic reprogramming in thyroid nodule patients, mainly characterized by the perturbation in GSH metabolism, the metabolism dysfunction of the TCA cycle, and disturbed metabolism of FAs, was represented by different levels of related 3-hydroxybutyric acid. Abnormal FAs metabolism is found to be related to PTC development and progression. Through this work, we confirmed that untargeted GC-MS-based metabolomics techniques could be employed to distinguish patients with thyroid nodules from healthy subjects, and more importantly it could probably discriminate malignancy from benignity. In consideration of the small sample size, this research could serve as a pilot study for future metabolomics studies that intend to solve the indeterminate samples problem.

## Data Availability Statement

All datasets generated for this study are included in the article/Supplementary Material.

## Ethics Statement

The study was conducted in accordance with the Declaration of Helsinki and was approved by the Institutional Review Board and Ethics Committee of Research Institute for Endocrine Sciences Shahid Beheshti University of Medical Sciences, Tehran, Iran. The patients/participants provided their written informed consent to participate in this study.

## Author Contributions

RA and MH were involved in the study concept and design. RA and SR provided the tools and patient specimens. RA, MG, and MS performed the experiments. RA, KH, and SR analyzed and interpreted the results and edited the manuscript. RA and MH organized the results and drafted the manuscript. MH approved the final version. All authors participated in the critical revision of the manuscript for important intellectual content.

## Conflict of Interest

The authors declare that the research was conducted in the absence of any commercial or financial relationships that could be construed as a potential conflict of interest.

## Footnotes

1 http://www.metaboanalyst.ca

## Abbreviations

FA: Fatty acid
FDR: False discovery rate
FNAB: Fine needle aspiration biopsy
GC-MS: Gas chromatography-mass spectrometry
MCFA: Median-chain fatty acid
MNG: Multinodular goiter
OPLS-DA: Orthogonal partial least squares-discriminant analysis
PCA: Principle component analysis
PTC: Papillary thyroid cancer
TCA: Tricarboxylic acid
VIP: Variable importance in the projection.
