# Supplementary material for: Plasma Metabolic Profiling of Human Thyroid Nodules by Gas Chromatography-Mass Spectrometry (GC-MS)-Based Untargeted Metabolomics
Source: Front Cell Dev Biol. 2020 Jun 16;8:385. doi: 10.3389/fcell.2020.00385 (PMC7308550; doi:10.3389/fcell.2020.00385)
Supplement: Supplementary file 1 [file Table_1.pdf]

Supplementary Tables

Supplementary Table S1. OPLS-DA models' parameters for plasma samples among three groups (healthy vs MNG vs PTC)

Supplementary Table S2: The mean values and standard deviations of the metabolite intensities

Supplementary Table S3. Significantly altered metabolites among three groups' classification (PTC, MNG, healthy) using one-way ANOVA analysis and Tukey's HSD test was used to analyses pairwise comparison (PTC vs H, MNG vs H and PTC vs MNG).

Supplementary Table S4. Parameters of OPLS-DA models for plasma samples between two groups separately.

Supplementary Table S5. Pathway analysis of PTC vs healthy

Supplementary Table S6. Pathway analysis of MNG vs healthy

Table S1 OPLS-DA models' parameters for plasma samples among three groups (healthy vs MNG vs PTC)

		**OPLS-DA Model**
**N**[a]		2 (*p*)[d] +3 (*O*)[e]
**R^2X(cum)**[b]		0.496
**R^2Y(cum)**[b]		0.871
**Q^2(cum)**[c]		0.617
**CV-ANOVA (p-value)**		1.51116e-11
**Permutation test**	**R^2Y (cum)**[b]	0.475
	**Q^2 (cum)**[c]	-0.579

a N: number of components.
b R2X (cum) and R2Y (cum) are the cumulative modeled variations in the X and Y matrix, respectively.
c Q2Y (cum) is the cumulative predicted variation in the Y matrix.
d P: Predictive component.
e O: Orthogonal component.

Table S2 The mean values and standard deviations of the metabolite intensities

Metabolites	Groups	Mean	Std. Deviation
Cysteine	Healthy	4.1949	2.8633
	PTC	-1.6558	1.9685
	MNG	-3.2773	2.2225
Cystine	Healthy	4.3614	3.4401
	PTC	-1.9444	2.8085
	MNG	-3.1428	1.9367
glutamic acid	Healthy	-4.8885	2.6587
	PTC	3.8403	4.0954
	MNG	1.5502	3.9171
sucrose	Healthy	-2.3376	2.3760
	PTC	-1.5254	2.1933
	MNG	4.7334	4.4750
arachidonic acid	Healthy	-2.4001	1.5029
	PTC	1.3583	2.2359
	MNG	1.3871	1.9531
oxoproline	Healthy	3.8927	2.1028
	PTC	-1.9375	4.2150
	MNG	-2.5651	2.7358
monomyristin	Healthy	-1.3864	2.0169
	PTC	-1.1241	2.3126
	MNG	3.0679	2.1283
glycine	Healthy	3.8349	2.9099
	PTC	-0.4748	4.2817
	MNG	-4.2298	3.8223
alpha-ketoglutarate	Healthy	-2.9757	1.4479
	PTC	1.5559	3.5257
	MNG	1.8720	2.9710
adenosine-5-monophosphate	Healthy	-2.3716	0.5509
	PTC	2.1179	3.4570
	MNG	0.4495	2.1274
linoleic acid	Healthy	-2.5976	2.2507
	PTC	1.0798	2.7325
	MNG	1.9648	2.7445
phenylalanine	Healthy	2.9867	2.4875
	PTC	-0.9121	3.6869
	MNG	-2.6503	2.9470
Serine	Healthy	2.6334	2.2949
	PTC	0.4657	4.0517
	MNG	-3.8447	3.7887
Oleic acide	Healthy	-2.8351	3.2773
	PTC	0.7064	2.8946
	MNG	2.7051	2.8921

2-monostearin	Healthy	-1.6479	2.0099
	PTC	-0.5044	2.7904
	MNG	2.6589	2.2138
threonine	Healthy	2.0397	2.6077
	PTC	0.4895	3.3412
	MNG	-3.1309	3.1254
palmitoleic acid	Healthy	-2.6180	2.9602
	PTC	1.2125	3.4169
	MNG	1.8327	2.6867
3-hydroxybutyric acid	Healthy	-4.4730	2.7934
	PTC	2.9877	6.3368
	MNG	2.0434	6.2446
methionine	Healthy	1.3385	1.9454
	PTC	0.2949	2.1399
	MNG	-2.0232	2.8333
Valine	Healthy	2.4230	2.8867
	PTC	-0.6424	3.9591
	MNG	-2.2658	4.1722
uracil	Healthy	-1.5409	2.0402
	PTC	1.5798	3.1369
	MNG	0.0501	3.0826
capric acid	Healthy	-2.2106	0.8928
	PTC	2.1067	5.9762
	MNG	0.2615	3.5751
lysine	Healthy	2.0848	2.9559
	PTC	-0.5757	4.9299
	MNG	-1.9223	3.2686
palmitic acid	Healthy	-1.3602	2.7557
	PTC	0.0612	2.2555
	MNG	1.6277	3.3341
malate	Healthy	-0.8027	1.4536
	PTC	1.2912	2.9605
	MNG	-0.5299	1.8623
2-hydroxybutanoic acid	Healthy	-1.9873	2.8771
	PTC	1.7662	3.7893
	MNG	0.3868	4.6621
inositol-4-monophosphate	Healthy	1.1599	2.1507
	PTC	-1.1133	2.3250
	MNG	-0.1279	2.6527
proline	Healthy	2.7777	4.7451
	PTC	-0.7138	6.1489
	MNG	-2.6245	6.3416

Table S3 Significantly altered metabolites among three groups' classification (PTC, MNG, healthy) using one-way ANOVA analysis and Tukey's HSD test was used to analyses pairwise comparison (PTC vs. H, MNG vs. H and PTC vs. MNG).

Metabolites	Subchemical classes	RT(min)	VIP	One-way ANOVA		Multiple comparisons (Tukey HSD)		
						PTC vs H	MNG vs H	PTC vs MNG
				P.value[a]	FDR	P.value[a]	P.value[a]	P.value[a]
cysteine	Amino acid	14.41	1.32	7.80E-13	4.68E-11	6.6851E-9	5.1051E-9	0.12
cystine	Amino acid	22.057	1.26	8.25E-11	2.47E-09	2.6352E-8	5.7718E-9	0.43
glutamic acid	Amino acid	15.262	1.49	1.32E-09	2.64E-08	6.6679E-9	0.0000006	0.15
phenylalanine	Amino acid	15.395	1.19	3.43E-06	1.62E-05	0.001	0.000004	0.22
serine	Amino acid	12.072	1.34	3.52E-06	1.62E-05	0.12	0.000002	0.001
oxoproline	Amino acid	14.143	1.26	3.79E-08	3.79E-07	0.000001	3.8688E-7	0.82
glycine	Amino acid	11.378	1.3	1.65E-07	1.24E-06	0.002	9.5189E-8	0.12
threonine	Amino acid	12.44	1.35	2.11E-05	7.89E-05	0.25	0.000015	0.003
methionine	Amino acid	14.091	1.19	0.000255	0.000804	0.34	0.000180	0.012
valine	Amino acid	10.039	0.99	0.00119	0.003571	0.32	0.001	0.4
lysine	Amino acid	18.6	0.89	0.008837	0.021997	0.87	0.008	0.55
Proline	Amino acid	11.191	0.92	0.020997	0.041994	0.14	0.019	0.59
arachidonic acid	Lipid	22.493	1.25	2.79E-08	3.35E-07	3.5517E-7	8.1325E-7	0.9
linoleic acid	Lipid	21.15	1.11	2.72E-06	1.49E-05	0.000127	0.000007	0.57
oleic acid	Lipid	21.192	1.15	5.08E-06	2.18E-05	0.002	0.000004	0.13
palmitic acid	Lipid	19.626	1.04	0.009246	0.021997	0.25	0.006	0.23
palmitoleic acid	Lipid	19.427	1.01	6.25E-05	0.000221	0.001	0.000189	0.82
capric acid	Lipid	13.204	0.97	0.006251	0.017049	0.004	0.17	0.37
monomyristin	Glycerolipid	22.748	1.49	7.23E-08	6.20E-07	0.9	3.2261E-7	0.000002
2-monostearin	Glycerolipid	25.647	1.31	5.52E-06	2.21E-05	0.294	0.000004	0.001
alpha-ketoglutarate	Keto acid	14.683	1.12	9.45E-07	6.30E-06	0.000013	0.000009	0.93
malate	dicarboxylic acid	13.731	1.3	0.0094734	0.021997	0.012	0.92	0.046
adenosine-5-monophosphate	nucleotide	30.576	1.44	1.09E-06	6.52E-06	7.0007E-7	0.002	0.10
uracil	nucleotide	11.68	1.07	0.003875	0.011072	0.003	0.21	0.24
inositol-4-monophosphate	Organooxygen	23.268	1.01	0.01529	0.032764	0.011	0.24	0.8
3-hydroxybutyric acid	Beta hydroxy acids	9.219	1.02	8.03E-05	0.000268	0.000157	0.002	0.85
2-hydroxybutanoic acid	Alpha hydroxy acids	8.716	0.96	0.010952	0.024338	0.009	0.15	0.53
Sucrose	Carbohydrate	25.148	1.6	9.39E-09	1.41E-07	0.6	3.1836E-8	6.1414E-7

ANOVA analysis of variance, MNG multinodular goiter, PTC papillary thyroid carcinoma, FDR false discovery rate, RT retention time

a P-values are from the one-way ANOVA and the Tukey post hoc test. P-value of < 0.05 was considered statistically significant

Table S4 Parameters of OPLS-DA models for plasma samples between two groups separately.

		OPLS-DA Models		
		**Healthy vs MNG**	**Healthy vs PTC**	**PTC vs MNG**
**N[a]**		1 (*P*)[d] +2 (*O*)[e]	1 (*P*)[d] +1 (*O*)[e]	1 (*P*)[d] +2 (*O*)[c]
**R^2X(cum)[b]**		0.486	0.314	0.357
**R^2Y(cum)[b]**		0.971	0.893	0.892
**Q^2(cum)[c]**		0.905	0.799	0.499
**CV-ANOVA (p-values)**		1.6687e-13	2.03403e-11	0.002
**Permutation Test**	**R^2Y(cum)[b]**	0.611	0.497	0.689
	**Q^2(cum)[c]**	-0588	-0.548	-0.812

a N: number of components.
b R2X (cum) and R2Y (cum) are the cumulative modeled variations in the X and Y matrix, respectively.
c Q2Y (cum) is the cumulative predicted variation in the Y matrix.
d P: Predictive component.
e O: Orthogonal component.

Table S5 Pathway analysis of PTC against healthy

Metabolic Pathways	Total Cmpd[a]	Hits[b]	Raw p[c]	FDR[d]	Impact
Glutathione metabolism	28	5	1.12E-13	5.83E-12	0.11891
Butanoate metabolism	15	4	1.76E-11	3.32E-10	0
Cysteine and methionine metabolism	33	4	2.70E-10	3.52E-09	0.22222
Histidine metabolism	16	1	9.57E-10	9.96E-09	0
Glyoxylate and dicarboxylate metabolism	32	8	3.07E-09	2.66E-08	0.33864
D-Glutamine and D-glutamate metabolism	6	3	1.19E-08	7.98E-08	0.5
Taurine and hypotaurine metabolism	8	1	1.43E-08	7.98E-08	0
Thiamine metabolism	7	1	1.43E-08	7.98E-08	0
Aminoacyl-tRNA biosynthesis	48	15	1.53E-08	7.98E-08	0.16667
Pantothenate and CoA biosynthesis	19	3	1.70E-08	8.01E-08	0
Arginine biosynthesis	14	5	4.35E-08	1.88E-07	0.17766
Arachidonic acid metabolism	36	1	1.36E-07	5.44E-07	0.3135
Alanine, aspartate and glutamate metabolism	28	7	2.61E-07	9.69E-07	0.36138
Nitrogen metabolism	6	2	4.30E-07	1.49E-06	0
Arginine and proline metabolism	38	3	6.52E-07	2.12E-06	0.27443
Biosynthesis of unsaturated fatty acids	36	5	5.71E-06	1.75E-05	0
Glycine, serine and threonine metabolism	33	5	8.54E-06	2.47E-05	0.48704
Synthesis and degradation of ketone bodies	5	1	1.69E-05	4.54E-05	0
Linoleic acid metabolism	5	1	1.75E-05	4.54E-05	1
beta-Alanine metabolism	21	1	0.000621	0.001537	0
Fatty acid biosynthesis	47	2	0.000868	0.002051	0.01473
Primary bile acid biosynthesis	46	2	0.001045	0.002362	0.05823
Purine metabolism	65	4	0.001985	0.004301	0.08068
Citrate cycle (TCA cycle)	20	5	0.00278	0.005783	0.2556
Phenylalanine, tyrosine and tryptophan biosynthesis	4	2	0.004155	0.008003	1
Phenylalanine metabolism	10	2	0.004155	0.008003	0.35714
Tryptophan metabolism	41	1	0.011024	0.020473	0.14305
Pyrimidine metabolism	39	2	0.01539	0.02674	0.0743
Fatty acid elongation	39	1	0.015941	0.02674	0
Fatty acid degradation	39	1	0.015941	0.02674	0
Propanoate metabolism	23	2	0.024061	0.0391	0
Sphingolipid metabolism	21	1	0.061399	0.092003	0
Lysine degradation	25	1	0.061925	0.092003	0
Biotin metabolism	10	1	0.061925	0.092003	0
Valine, leucine and isoleucine biosynthesis	8	3	0.068438	0.098854	0
Ubiquinone and other terpenoid-quinone biosynthesis	9	1	0.072727	0.10221	0
Valine, leucine and isoleucine degradation	40	2	0.076893	0.10307	0

Pyruvate metabolism	22	3	0.078271	0.10307	0.0311
Tyrosine metabolism	42	2	0.079283	0.10307	0.16435
Steroid biosynthesis	42	1	0.16768	0.20761	0.0282
Steroid hormone biosynthesis	85	1	0.16768	0.20761	0.00528
Glycolysis / Gluconeogenesis	26	1	0.34957	0.42274	0
Selenocompound metabolism	20	1	0.43252	0.51116	0
Glycerolipid metabolism	16	2	0.53647	0.61992	0.33022
Starch and sucrose metabolism	18	2	0.66846	0.74201	0.47093
Inositol phosphate metabolism	30	1	0.6992	0.74201	0.12939
Phosphatidylinositol signaling system	28	1	0.6992	0.74201	0.03736
Ascorbate and aldarate metabolism	8	1	0.6992	0.74201	0
Galactose metabolism	27	5	0.77616	0.79724	0.07387
Pentose phosphate pathway	22	1	0.78191	0.79724	0

[a]Total Cmpd: total number of compounds in the pathway
[b]Hit: actually matched number from the data
[c] Raw p : p value calculated from the enrichment analysis
[d]FDR: p value adjusted using False Discovery Rate
PTC, papillary thyroid cancer; FDR, false discovery rate

Table S6 Pathway analysis of MNG against healthy

Metabolic Pathways	Total Cmpd[a]	Hits[b]	Raw p[c]	FDR[d]	Impact
Glutathione metabolism	28	5	2.57E-15	1.34E-13	0.11891
Glyoxylate and dicarboxylate metabolism	32	8	4.67E-13	1.21E-11	0.33864
Cysteine and methionine metabolism	33	4	1.77E-12	2.44E-11	0.22222
Aminoacyl-tRNA biosynthesis	48	15	1.88E-12	2.44E-11	0.16667
Glycine, serine and threonine metabolism	33	5	1.45E-10	1.26E-09	0.48704
Taurine and hypotaurine metabolism	8	1	1.80E-09	1.17E-08	0
Thiamine metabolism	7	1	1.80E-09	1.17E-08	0
Arginine biosynthesis	14	5	8.04E-09	4.64E-08	0.17766
Arachidonic acid metabolism	36	1	9.19E-09	4.78E-08	0.3135
Pantothenate and CoA biosynthesis	19	3	2.56E-08	1.21E-07	0
Butanoate metabolism	15	4	2.98E-08	1.29E-07	0
D-Glutamine and D-glutamate metabolism	6	3	3.45E-08	1.38E-07	0.5
Biosynthesis of unsaturated fatty acids	36	5	1.03E-07	3.81E-07	0
Histidine metabolism	16	1	1.11E-07	3.85E-07	0
Linoleic acid metabolism	5	1	4.44E-07	1.44E-06	1
Primary bile acid biosynthesis	46	2	5.05E-07	1.49E-06	0.05823
Arginine and proline metabolism	38	3	5.15E-07	1.49E-06	0.27443
Sphingolipid metabolism	21	1	1.08E-06	2.95E-06	0
Nitrogen metabolism	6	2	3.71E-06	9.64E-06	0
Fatty acid biosynthesis	47	2	2.04E-05	5.04E-05	0.01473
Alanine, aspartate and glutamate metabolism	28	7	5.91E-05	0.00014	0.36138
Valine, leucine and isoleucine biosynthesis	8	3	8.37E-05	0.000189	0
Synthesis and degradation of ketone bodies	5	1	8.95E-05	0.000194	0
Galactose metabolism	27	5	9.52E-05	0.000198	0.07387
Starch and sucrose metabolism	18	2	0.000108	0.000217	0.47093
Phenylalanine, tyrosine and tryptophan biosynthesis	4	2	0.000172	0.000304	1
Phenylalanine metabolism	10	2	0.000172	0.000304	0.35714
Fatty acid elongation	39	1	0.000175	0.000304	0
Fatty acid degradation	39	1	0.000175	0.000304	0
Citrate cycle (TCA cycle)	20	5	0.002926	0.004909	0.2556
Valine, leucine and isoleucine degradation	40	2	0.003029	0.004916	0
Lysine degradation	25	1	0.003214	0.004916	0
Biotin metabolism	10	1	0.003214	0.004916	0
Glycerolipid metabolism	16	2	0.012611	0.018736	0.33022
Tyrosine metabolism	42	2	0.039147	0.056545	0.16435
Purine metabolism	65	4	0.052541	0.073841	0.08068
Tryptophan metabolism	41	1	0.056911	0.077835	0.14305
Ubiquinone and other terpenoid-quinone biosynthesis	9	1	0.058376	0.077835	0

beta-Alanine metabolism	21	1	0.061634	0.080124	0
Propanoate metabolism	23	2	0.24233	0.30735	0
Pyruvate metabolism	22	3	0.25888	0.32052	0.0311
Pyrimidine metabolism	39	2	0.42549	0.50286	0.0743
Selenocompound metabolism	20	1	0.4255	0.50286	0
Neomycin, kanamycin and gentamicin biosynthesis	2	1	0.46684	0.50716	0
Steroid biosynthesis	42	1	0.47266	0.50716	0.0282
Steroid hormone biosynthesis	85	1	0.47266	0.50716	0.00528
Inositol phosphate metabolism	30	1	0.48766	0.50716	0.12939
Phosphatidylinositol signaling system	28	1	0.48766	0.50716	0.03736
Ascorbate and aldarate metabolism	8	1	0.48766	0.50716	0
Glycolysis / Gluconeogenesis	26	1	0.55196	0.56279	0
Pentose phosphate pathway	22	1	0.67679	0.67679	0

[a]Total Cmpd: total number of compounds in the pathway,
[b]Hit: actually matched number from the data,
[c] Raw p : p value calculated from the enrichment analysis,
[d]FDR: p value adjusted using False Discovery Rate

MNG, multinodular goiter; FDR, false discovery rate
