# Supplementary material for: Plasma Metabolic Profiling of Human Thyroid Nodules by Gas Chromatography-Mass Spectrometry (GC-MS)-Based Untargeted Metabolomics
Source: Front Cell Dev Biol. 2020 Jun 16;8:385. doi: 10.3389/fcell.2020.00385 (PMC7308550; doi:10.3389/fcell.2020.00385)
Supplement: Supplementary file 2 [file Data_Sheet_1.pdf]

Supplementary Figures

Supplementary Figure S1. The GC–Q-MS total ion current (TIC) chromatograms of plasma from a healthy subject.

Supplementary Figure S2. The principal component analysis **(**PCA) scores plot obtained from GC-Q-MS data of all samples.

Supplementary Figure S3. Validation plot of a 100 permutation test for OPLS-DA model built for plasma samples among three groups.

Supplementary Figure S4. Validation plot of 100 permutation tests for OPLS-DA models built for plasma samples between two groups separately.

Supplementary Figure S5. Venn diagram analysis of discriminant metabolites in different groups, (blue) PTC vs Healthy, (yellow) MNG vs Healthy and (green) MNG vs PTC.

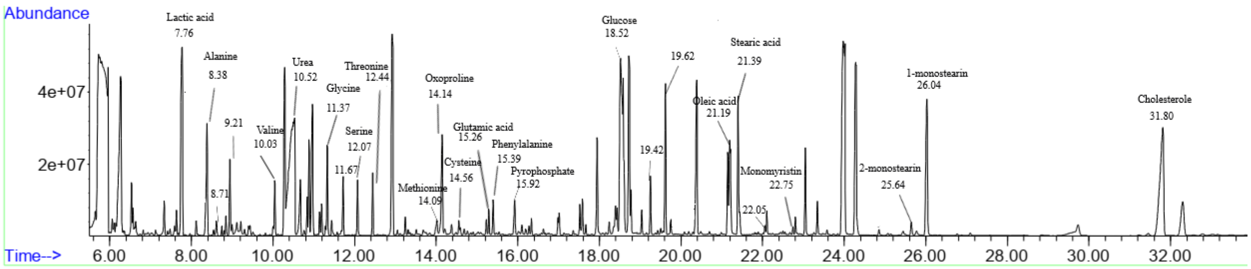

Figure S1 The GC–Q-MS total ion current (TIC) chromatograms of plasma from a healthy subject

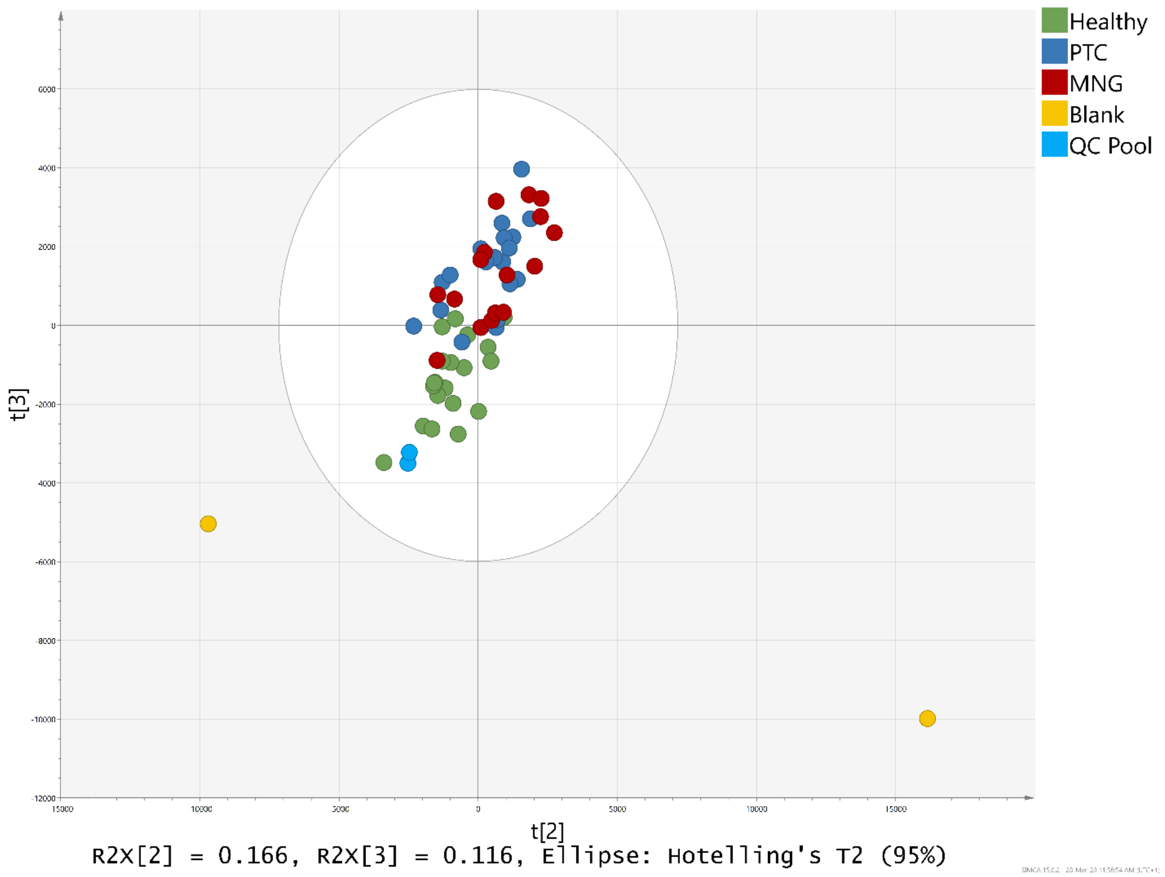

Figure S2: The principal component analysis **(**PCA) scores plot obtained from GC-Q-MS data of all samples. All quality control (QC) samples are found to cluster in the PCA plot.

PTC; papillary thyroid cancer, MNG; multi nodular goiter, QC; quality control

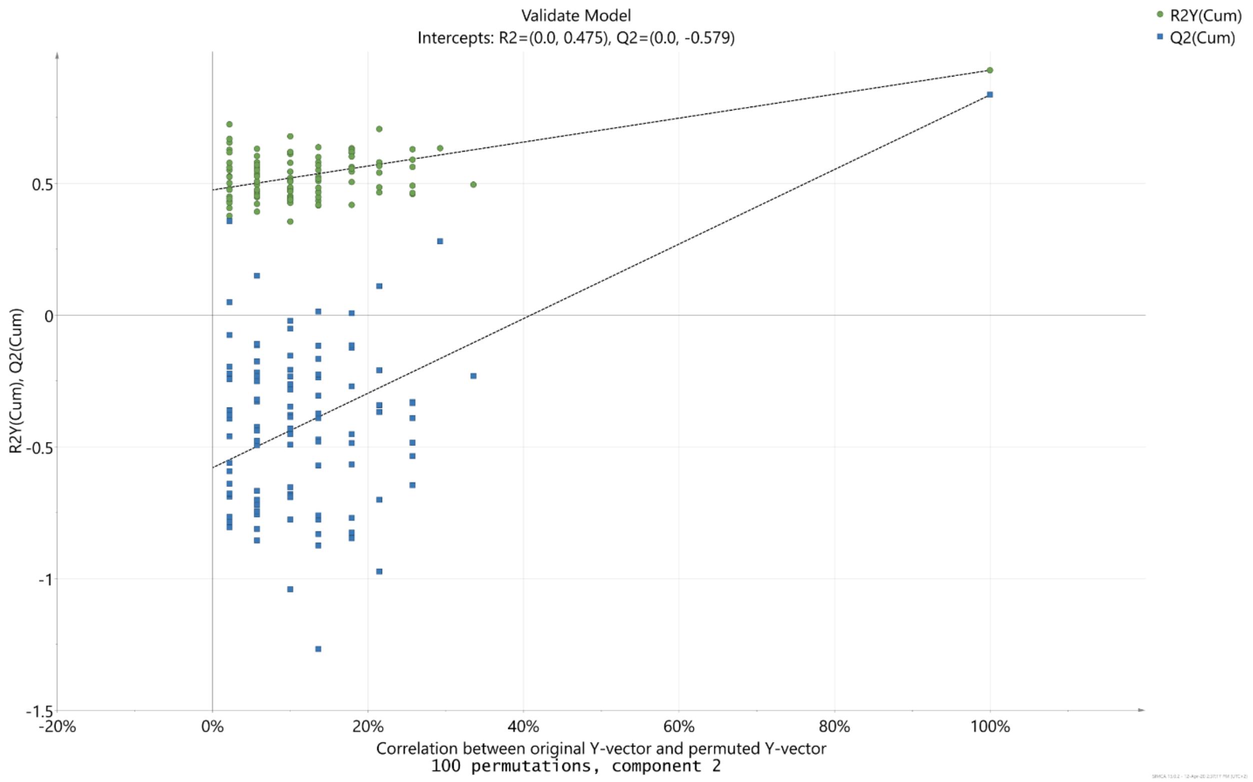

Figure S3: Validation plot of a 100 permutation test for OPLS-DA model built for plasma samples among three groups. Low value of R2Y and Q2-intercepts at 0.475 and -0.579 indicating a valid model.

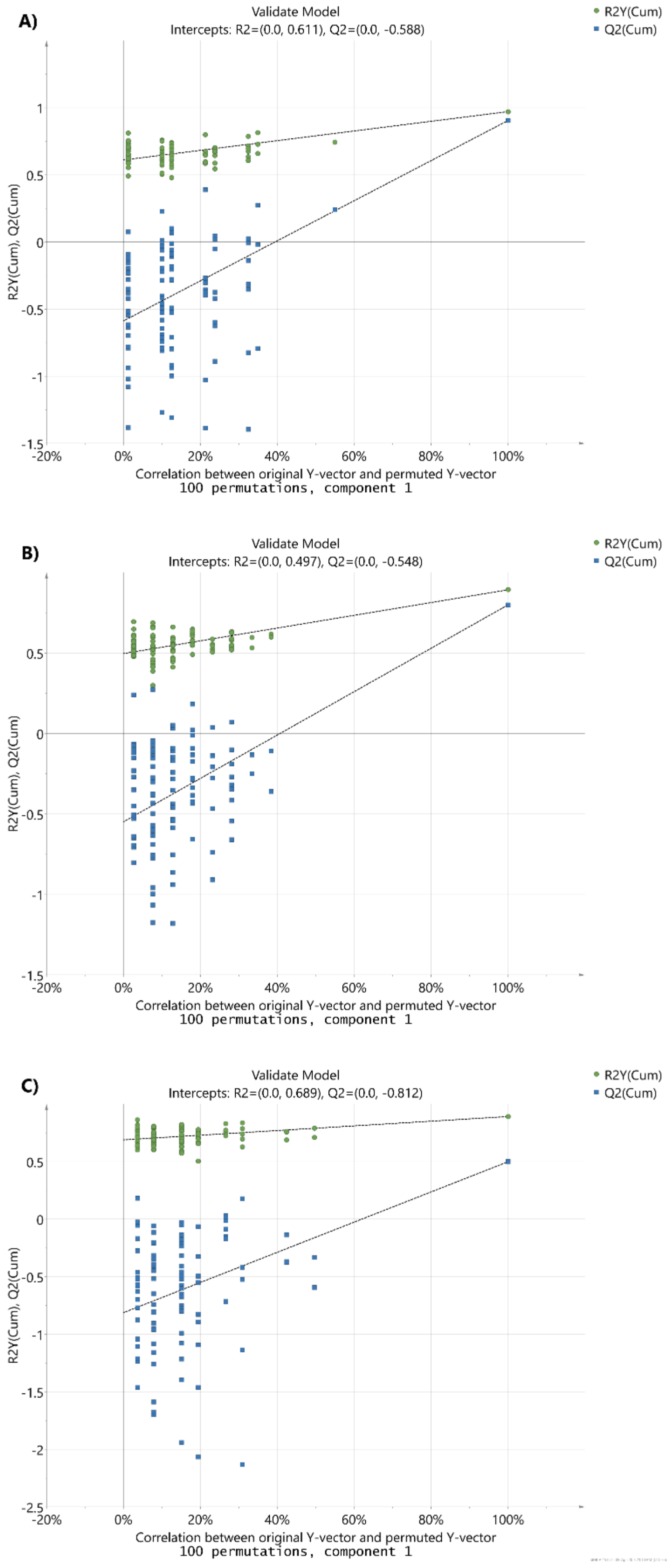

Figure S4. Validation plot of 100 permutation tests for OPLS-DA models built for plasma samples **(A)** MNG vs healthy, **(B)** PTC vs healthy and **(C)** PTC vs MNG. The 3 plots assure that the 3 models were well-preserved against overfitting.

MNG; multi nodular goiter, PTC; papillary thyroid cancer

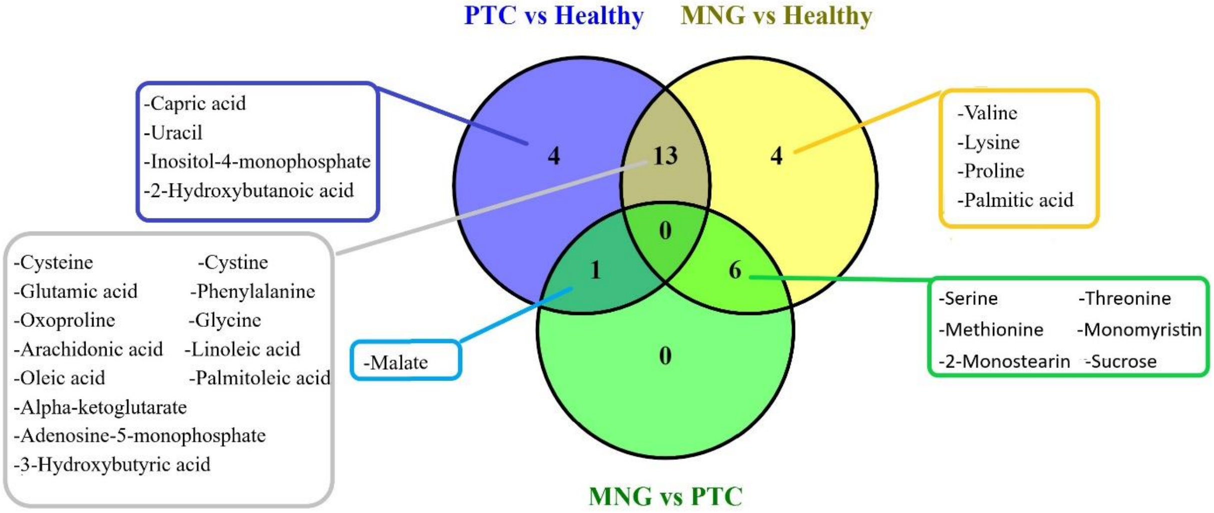

Figure S5 Venn diagram analysis of discriminant metabolites in different groups, (blue) PTC vs Healthy, (yellow) MNG vs Healthy and (green) MNG vs PTC.

MNG; multi nodular goiter, PTC; papillary thyroid cancer
